# Severe rectal burn induced by hot normal saline enema: a case report

**DOI:** 10.1093/gastro/goac027

**Published:** 2022-06-14

**Authors:** Xiaoming Zhu, Siyuan Jiang, Chen Wang, Haifeng Gong, Wei Zhang

**Affiliations:** Department of Colorectal Surgery, Changhai Hospital, Shanghai, P. R. China; Department of Colorectal Surgery, Changhai Hospital, Shanghai, P. R. China; Department of Colorectal Surgery, Changhai Hospital, Shanghai, P. R. China; Department of Colorectal Surgery, Changhai Hospital, Shanghai, P. R. China; Department of Colorectal Surgery, Changhai Hospital, Shanghai, P. R. China

## Case report

A 44-year-old woman was admitted to our emergency room with a diagnose of rectal burn due to an accidental hot normal saline enema before oophorocystectomy in a local hospital 8 days previously. She reported hypogastralgia and burning pain on the buttocks immediately when the enema was conducted. The enema was stopped instantly and she was treated with fasting, antibiotics, glucocorticoids, and parenteral nutrition for a week. However, the above symptoms did not resolve.

**Figure 1.**
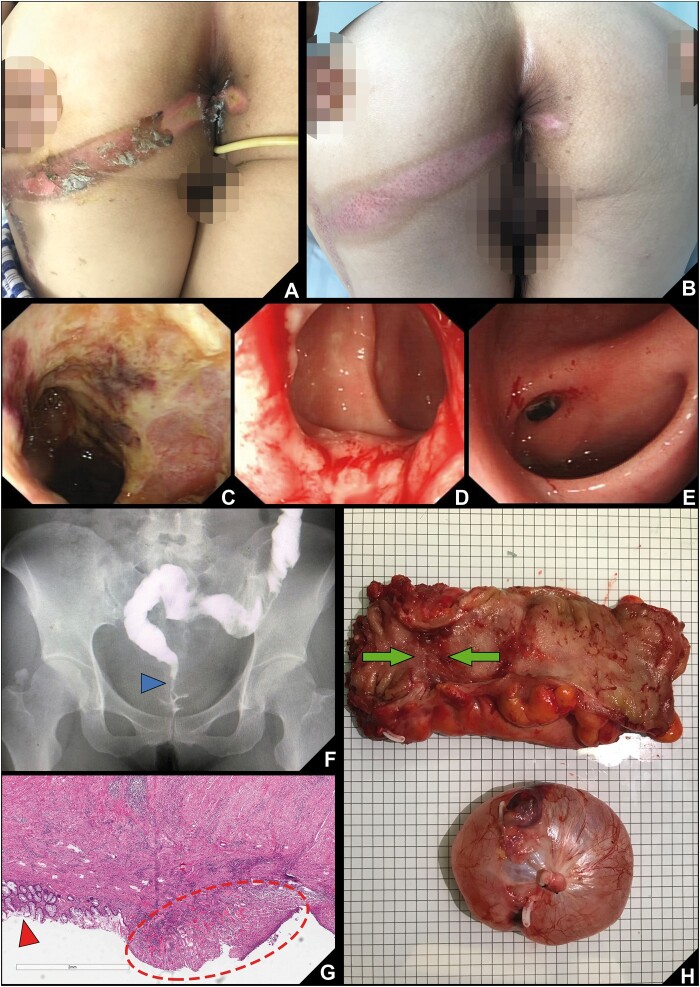
Clinical data of a patient with severe rectal burn induced by a hot normal saline enema. (A) Wounds on the buttocks before treatment in our hospital. (B) Wounds after 4 weeks of treatment. (C) Colonoscopic appearance of the rectum before treatment in our hospital. (D) The colonoscope could be advanced to the sigmoid colon at 4 weeks of treatment. (E) At 2 months after injury, an impassable stricture was found 5 cm above the anal verge. (F) Iodine angiography of lower digestive tract shows the stricture on the rectum (blue triangle). (G) Histologic examination (×40 HPF (high power field)). The red triangle shows normal mucosa whereas the red dotted circle shows chronic inflammation and fibrosis of the mucosa. (H) Specimen of the rectum and ovarian cyst (green arrows show the stricture of the rectum). (A colour version of this figure appears in the online version of this article.)

Second-degree burn scars were seen on both buttocks (Figure 1A). Digital rectal examination revealed the anal function was normal, but the rectal wall was rough without mucosal folds. In addition, colonoscopy revealed severe edema, necrosis, and fibrin exudation around the rectal wall. However, the colonoscope could not be advanced beyond 10 cm above the anal verge due to luminal narrowing (Figure 1C).

The patient was admitted and treated with a combination of fasting, proton-pump inhibitors, antibiotics, glucocorticoids, and parenteral nutrition. Sulfadiazine Zinc Silver cream was applied on the buttock wounds. Meanwhile, a retention enema was performed twice a day using 200 mL of Kangfuxin solution to accelerate rectal mucosa healing. After treatment for 1 week, hypogastralgia and hematochezia were gradually resolved, and she could have a liquid diet. After 2 weeks of treatment, she could have a semi-liquid diet. By Week 4 of treatment, the buttock wounds had almost healed (Figure 1B). Colonoscopy revealed a nodular granulation tissue with hyperemia and edema on the mucosa 3–10 cm above the dentate line. Although the lumina was still relatively narrow, the colonoscope could be advanced to the sigmoid colon (Figure 1D). Hypogastralgia and hematochezia had disappeared and she could pass loose stool. The patient was discharged on Day 30 after admission. She continued the Kangfuxin solution retention enema at home.

About 1 month after discharge, she reported symptoms of frequent defecation, tenesmus, hematochezia, and occasional abdominal pain. She underwent colonoscopy that revealed an impassable stricture 5 cm above the anal verge (Figure 1E). Iodine angiography of the lower digestive tract showed that there was a stricture on the rectum (Figure 1F). Finally, we performed laparoscopic proctectomy (low anterior resection) with temporary terminal ileostomy and right oophorocystectomy 2 months after her injury (Figure 1G and H). The ileal diversion was buried 2 months later.

## Discussion

Rectal burn is quite rare. In Western countries, it is mainly caused by a hot coffee or hydrogen peroxide enema in patients with constipation, which causes thermal or chemical injury to the rectal mucosa [1, 2]. In rural China, rectal burn is mainly caused by an enema using folk remedies. In rare cases, a hot normal saline enema can cause iatrogenic damage [3]. Here we report a case of rectal burn due to a hot normal saline enema. This type of enema can cause thermal injury to the rectum that may lead to proctitis, pelvic infection, rectal perforation, and rectal stricture. The symptoms include hypogastralgia, hematochezia, tenesmus, and anxiety.

The current literature about rectal burn only focuses on its manifestations, treatment, and outcome rather than its pathophysiological changes. The severity of a scald of the skin is influenced by temperature of the hydrotherm, the contact time, and the extent of the burns [4]. Therefore, we believe the temperature and dosage of saline, contact time with the rectum, and patient’s position during the enema will affect the severity of a rectal burn. According to the Chinese scald grading and the grading system of inhalation injury (derived from findings at initial bronchoscopy and based on the Abbreviated Injury Score) [3, 5], we propose that the rectal burn can be divided into three types. Type 1: simple mucosa burn. Rectal mucosa presents with hyperemia, edema, exudation, and local necrosis, which is similar to ischemic proctitis. This type can be treated conservatively and leaves a faint scar [6]. Type 2: muscular layer burn. It is characterized by sever edema, exudation, and local ulceration of the mucosa. Other symptoms include hematochezia, tenesmus, abdominal pain, and fever. Conservative treatment is preferred if there are no severe systemic symptoms. Scars always remain on the healed rectum, but the degree of scarring varies depending on the extent of the burn and outcome of conservative treatment [7, 8]. Type 3: full-thickness burn. It is the most severe type that presents with rectal perforation and pelvic and systemic infection. Patients also suffer from the local symptoms of Types 1 and 2 and they have to accept colostomy in the preliminary stage to create conditions for rectum healing. Unfortunately, the healing process is often accompanied by permanent rectal stricture that eventually requires proctectomy [9, 10].

Based on this classification, our patient had a Type 2 rectal burn. Therefore, she was treated with a combination of fasting, proton-pump inhibitors, antibiotics, glucocorticoids, and parenteral nutrition. In addition, the Kangfuxin solution was used for a retention enema to accelerate rectal mucosa healing and this has never been reported before. The initial conservative treatment substantially improved her condition and she could pass out loose stool fairly well for a period of time. Unfortunately, severe scarring caused a rectal stricture, which warranted proctectomy.

In conclusion, a rectal burn caused by a hot normal saline enema is quite rare. The outcome of conservative treatment depends on the degree of burn and surgical resection of the involved segment should be the last choice. Based on our classification, we should conduct individualized treatments for different types of rectal burns. A better understanding of its pathophysiological process is still needed.

## Authors’ Contributions

X.Z., S.J., and C.W. collected the data and drafted the manuscript. H.G. and W.Z. made critical revisions related to important intellectual content of the manuscript. All authors have read and approved the final version of the manuscript.

## Funding

This work was supported by the 234 Discipline Climbing Program of the First Affiliated Hospital of Naval Medical University [grant number 2019YXK032].
